# Accurate, non-destructive, and high-throughput age estimation for Golden perch (*Macquaria ambigua* spp.) using DNA methylation

**DOI:** 10.1038/s41598-023-36773-2

**Published:** 2023-06-12

**Authors:** Benjamin Mayne, Tom Espinoza, David A. Crook, Chloe Anderson, Darren Korbie, Jonathan C. Marshall, Mark J. Kennard, Doug J. Harding, Gavin L. Butler, Brien Roberts, Josh Whiley, Sharon Marshall

**Affiliations:** 1 Environomics Future Science Platform, Commonwealth Scientific and Industrial Research Organisation (CSIRO), Indian Ocean Marine Research Centre, Crawley, WA Australia; 2 Department of Regional Development, Manufacturing and Water, Brisbane, QLD Australia; 3 Department of Primary Industries, Narrandera Fisheries Centre, Narrandera, NSW Australia; 4 Australian Institute for Bioengineering and Nanotechnology, The University of Queensland, Brisbane, QLD Australia; 5 Queensland Department of Environment and Science, Brisbane, QLD Australia; 6 Australian Rivers Institute and Griffith School of Environment and Science, Griffith University, Nathan, QLD 4111 Australia; 7 NSW Department of Primary Industries (Fisheries), Grafton, NSW Australia; 8 Fisheries Division, Department of Industry, Tourism and Trade, Darwin, NT Australia

**Keywords:** Bioinformatics, Genomic analysis, Environmental sciences, Biomarkers

## Abstract

Age structure information of animal populations is fundamental to their conservation and management. In fisheries, age is routinely obtained by counting daily or annual increments in calcified structures (e.g., otoliths) which requires lethal sampling. Recently, DNA methylation has been shown to estimate age using DNA extracted from fin tissue without the need to kill the fish. In this study we used conserved known age-associated sites from the zebrafish (*Danio rerio*) genome to predict the age of golden perch (*Macquaria ambigua*), a large-bodied native fish from eastern Australia. Individuals aged using validated otolith techniques from across the species’ distribution were used to calibrate three epigenetic clocks. One clock was calibrated using daily (daily clock) and another with annual (annual clock) otolith increment counts, respectively. A third used both daily and annual increments (universal clock). We found a high correlation between the otolith and epigenetic age (Pearson correlation > 0.94) across all clocks. The median absolute error was 2.4 days in the daily clock, 184.6 days in the annual clock, and 74.5 days in the universal clock. Our study demonstrates the emerging utility of epigenetic clocks as non-lethal and high-throughput tools for obtaining age estimates to support the management of fish populations and fisheries.

## Introduction

Information on the age structure of animal populations is fundamental to their conservation and management, providing the basis for enumeration of mortality and recruitment rates, and other key demographic parameters^1^. In fisheries science, age estimates are routinely obtained by counting increments in calcified structures, such as otoliths (ear stones), which requires lethal sampling^2^. However, it has been shown that DNA methylation, an epigenetic modification is predictive of age^3–5^. Epigenetic clocks which use DNA methylation at cytosine-phosphate-guanosine (CpG) sites to predict age have now demonstrated the ability to predict age across a broad spectrum of wild animals using non-lethal, high-throughput, and cost-effective techniques^6–8^.

Epigenetic clocks have been made across a broad range of fish species^4,5,9,10^. There is increasing interest in using epigenetic clocks in fisheries management and conservation^11,12^. The advantage of an epigenetic clock is the ability to predict age from a somatic tissue, making it a non-lethal method. Increasing concern about the sustainability of freshwater and marine fisheries, widespread fish population declines, and growing species extinction rates^13,14^, means that non-lethal fish sampling for scientific research is growing in importance globally. Moreover, increasing awareness of the need for ethical treatment of fish by scientists, managers and the public makes non-lethal sampling highly desirable^15^.

Golden perch (*Macquaria ambigua*) is a large-bodied, native fish naturally distributed throughout the Murray-Darling, Lake Eyre, Fitzroy River, and Bulloo River basins in eastern Australia. Golden perch are capable of large-scale migrations of > 1000 km^16^. The taxonomy of the golden perch complex has not yet been fully resolved; however, the Murray-Darling (*M. ambigua ambigua*) and the Fitzroy River (*M. ambigua oriens)* lineages are widely recognised as distinct subspecies^17^. Golden perch are relatively long-lived (up to 27 years) and commonly grow to 500 mm total length and 5 kg in weight^18^. Although the species historically supported viable commercial fisheries, commercial harvest has been drastically scaled-back in recent decades and is currently only permitted in restricted areas of South Australia^19^. Golden perch are highly valued by recreational fishers and support important traditional fisheries^20,21^. Due to their economic, social, and cultural value, golden perch have been the focus of extensive management activity. This includes the large-scale stocking of hatchery-produced fingerlings to improve populations, restoration of fish passage, and the provision of environmental flows in rivers to enhance spawning and recruitment and initiate migratory behaviours^22–24^.

Information on the ecology of golden perch populations has been central to the development of conservation and management strategies for the species over many decades: this includes studies of age-length relationships, growth, longevity, spawning and recruitment dynamics, outcomes of stocking, movement and migration patterns, and stock assessment^19,25–34^. To date, age data has been generated via counts of annual or daily increments in otoliths. The use of annual increment counts for age estimation in golden perch was evaluated by Anderson et al.^35^, who examined marginal increments to confirm that opaque bands were deposited in the otolith structure around austral spring each year^35^. Stuart^36^, used an independent methodology to further validate estimated annual ages from increment counts by analysing the otoliths of known-age fish from a lake stocked > 20 years previously^36^. Counts of daily increments in the otoliths of larval and early daily golden perch have also been validated as a means of deriving age estimates at a daily time scale^37^. Although the use of otolith increments for ageing of golden perch is well-established and highly reliable, a major disadvantage of this approach is the requirement to kill fish to obtain their otoliths for analysis. In a commercial fishery setting, where access to lethally harvested fish is generally available, this is not problematic. However, for highly valued species like golden perch which are not commercially harvested across most of their range, the requirement for sampling can place severe limitations on the ability to obtain high-quality age information.

In this study, we develop an epigenetic clock for the non-lethal age prediction of golden perch. Paired otolith and fin tissue samples from *M. ambigua ambigua* (Murray-Darling Basin) and *M. ambigua oriens* (Fitzroy Basin) were used to calibrate and validate the model. We constructed three separate models using daily, annual, and combined aged otoliths. Our study demonstrates the applicability of using epigenetic clocks in meeting the challenges of wildlife research and management.

## Methods

### Statement of ethical approval

Golden perch used in the study were from three broad-scale regions from across its range. Specific permits were obtained for each region. All methods are reported in accordance with ARRIVE guidelines and approved by the appropriate institutional licensing committees from the Queensland Department of Primary Industries and Fisheries (Animal Ethics Committee Permits: CA 2008/09/296, SA 2020/10/760, General Fisheries Permits: 89474, 198642, 153301, and 191663), Griffith University Animal Ethics Committee permit (GU Ref. No: ENV/02/21/AEC), NSW Department of Primary Industries Animal Care and Ethics permit (DPI Ref. No. 14/10) and DPI Fisheries Section 37 Scientific Collection permit (Permit No. P01/0059(A)-4.0). Otoliths were removed, dried, and stored in individual packets, with a tissue sample collected from the dorsal or caudal fin preserved in ethanol for genetic analysis. A total 276 paired otolith and genetic samples were used in this study (Supplementary Table 1).

### Otolith preparation and analyses

Sagittal otoliths used for daily aging were extracted from each fish (9.61–190 mm length range) using a dissecting microscope. Otoliths from fish > 25 mm length were cleaned and dried, then mounted sulcus side up on a glass slide with Crystalbond 509 thermopolymer. Mounted otoliths were then polished, using 400 grit sandpaper to expose the otolith core and finished using 30-micron and 3-micron diamond lapping film, as described in Stevenson and Campana^38^. Otoliths removed from fish < 25 mm length, were placed uncleaned on a concave slide with a drop of immersion oil and covered with a slide cover. An Olympus BX53 compound microscope was then used to take high-definition images of each otolith at 100–200 × magnification. Images were then imported into image editing software (paint.net v. 4.2.10), which was used to mark the daily incremental rings (Supplementary Fig. 1A). Where necessary, multiple images were stitched together using different focal lengths to allow all rings to be viewed. To minimise bias, otolith ring counting was completed without knowledge of the size and expected age of each individual fish. Age estimates was verified using a subsample of 23 otoliths aged by two different readers. Average Percent Error was 4.86%^39^. Five days were added to the daily age estimates as this equates to the average duration between spawning and the formation of the first daily ring^37^.

Sagittal otoliths for annual aging were dissected from each fish, embedded in 2-part epoxy resin and transversely sectioned to a thickness of ~ 300 µm through the primordium using an Isomet Low Speed Saw (Buehler, IL, USA). Sections were then mounted on glass microscopic slides using resin and photographed using transmitted light at × 16 or × 20 magnifications. Images were viewed using image analysis software (Leica Application Suite, v. 4.2) and the age of each fish was estimated by counting annuli along a radius from the primordium to the outer edge of the dorsal lobe (Supplementary Fig. 1B). Marginal increments were visually classified as narrow, intermediate, or wide^40^. To account for variability in the seasonal timing of increment formation among individual fish, increment counts were adjusted based on the marginal increment status and month of capture^41^. Otoliths with a wide marginal increment that were captured within a three-month period after the putative increment formation date were assumed to be ‘late’ in forming an annual increment; thus, one year was added to the increment count to estimate biological age^35^. Average Percent Error for readers is 3.9%^42^. Similarly, otoliths with a narrow marginal increment captured less than three months prior to October 1 were assumed to be ‘early’ in forming an increment, therefore one year was subtracted from the increment count.

### Genome pairwise alignment

In a previous study, reduced representation bisulfite sequencing (RRBS) was used as a genome wide method to identify age associated CpG sites in zebrafish (*Danio rerio*)^4^. In total, DNA methylation at 1,311 CpG sites were found to have a significant positive or negative correlation with age (Pearson correlation, p-value < 0.05). Age associated CpG sites in the golden perch genome (assembly GOP001_1.1) were identified by genome pairwise alignment with known age associated CpG sites in the zebrafish genome (danRer10). Genome pairwise alignment was carried out between the golden perch and zebrafish genome with LASTZ with the following options: *–notransition –step* = *20 –nogapped*^43^. Of the 1,311 known age associated CpG sites in zebrafish, 40 were conserved in golden perch.

### Measuring DNA methylation through multiplex PCR

Primers for multiplex PCR were designed using PrimerSuite (www.primer-suite.com), with an annealing temperature of 55 °C, 125-155 bp amplicon range, one pool of primers, unmethylated cytosines in the primer sequences, and the two fusion sequences for subsequent barcoding (gacatggttctaca and cagagacttggtct)^44^. These fusion sequences were chosen to fit the Fluidigm 384 barcodes (Fluidigm Cat# 100–4876). Of the 40 CpG sites found to be conserved between zebrafish and golden perch, 31 were able to be included in the multiplex PCR primer design (Supplementary Table 2). The software excluded 9 CpG sites due to the high probability of factors such as primer dimers and interactions between primers for one primer pool. Although the target is 31 CpG sites, the amplicons contain neighbouring CpG sites. We decided to include neighbouring CpG sites in the model as they may have the potential to improve the age prediction.

DNA from the samples (Supplementary Table 1) were extracted using the DNeasy Blood & Tissue Kit (Qiagen, Cat. 69504) following the manufacturer’s protocol. A total of 150 ng of DNA was bisulfite-treated using a modified version of a publicly available protocol (Supplementary Material 1)^45^. Individual primers (Supplementary Table 2) were tested in singleplex with annealing temperatures ranging between 56–60 °C (Promega, Cat. M5006). Primer pairs that produced a single band and no primer dimers were included in the multiplex. Primers included in the multiplex PCR were also tested with total primer concentrations 0.6–1.2 µM and annealing temperatures of 56 – 60 °C. A final primer concentration of 0.8 µM annealed at 56 °C was used for the final multiplex reaction (Supplementary Tables 3 and 4). Barcoding of each sample was carried out with Fluidigm 384 barcodes (Supplementary Tables 5 and 6) and were pooled together equally by volume. DNA sequencing was carried out with an Illumina MiSeq, 300 cycle, and generated 30 × coverage.

### Sequencing data and model generation

Sequencing data was clipped with seqkit v1.2 by 15 bp at both the 5' and 3' ends^46^. Trimmed reads were then aligned to the golden perch genome (assembly GOP001_1.1) with Bismark v0.20.0 and Bowtie2 v2.4.4 as an aligner with default parameters^47,48^. Methylation calling was carried out with Bismark’s bismark_methylation_extractor using the default parameters. After methylation calling, CpG sites with < 100 reads in a sample were removed from the data set.

Samples were randomly assigned but stratified by age using the caret R package^49^ into either a training data set to calibrate the model (70% of samples) or a testing data set to independently validate the model (30% of samples). Samples were stratified by age to prevent a bias of an age class being in one data set. The fish ages for the samples that were used to calibrate the model were either daily or annual ages derived from their otoliths (Supplementary Table 1). To make best use of the available data, three independent models were generated. The first was a daily clock that was only calibrated with samples that had daily age predictions from otoliths; the second was an annual clock that was only calibrated with samples that had annual age predictions from otoliths; and the third a universal model, that incorporated all samples. An elastic net regression model with a tenfold cross validation and the alpha parameter set to 0.5 was used to identify the best CpG sites as predictors of age^50^. An elastic net regression model is a commonly used algorithm used in epigenetic ageing studies as it can identify the best performing predictors from potentially 1000 s of candidates^50,51^. Although we have already narrowed it down to 31 CpG sites, there were potentially neighbouring CpG sites that may be specific to golden perch and may improve the epigenetic clock. A separate elastic net regression model was used for each clock to make a specific model for each set of samples. A Pearson correlation was used to determine the precision of the model by using the correlation between the otolith and predicted epigenetic ages. The median absolute error rate between the training and testing datasets was used to determine if there was any potential overfitting. Relative error was used to determine the overall performance of the model. Relative error was determined by taking the absolute error and dividing it by the otolith age. A Pearson correlation was also used with age versus relative error for each sample to determine if the model was biased towards younger or older aged samples. If a statistical difference was observed between the training and testing datasets in either Pearson correlation, absolute or relative error rates, it would suggest potential overfitting. Student t-tests and Kolmogorov–Smirnov (KS) were conducted to determine if there was a statistical difference between the training and testing datasets. All analyses were undertaken in R v4.0.4^52^ and R scripts for the elastic net are provided in the supplementary.

## Results

### Otolith ageing

**Figure 1:**
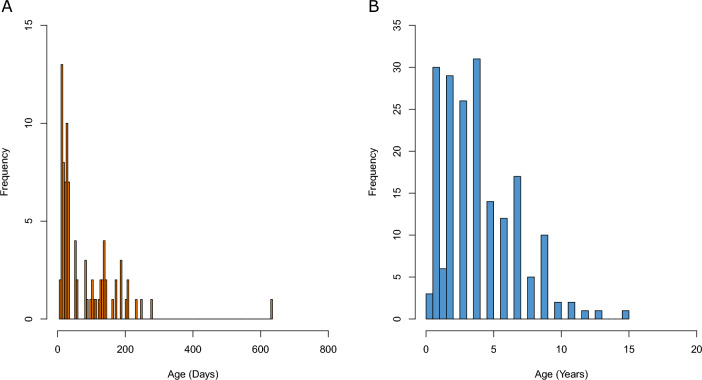
Histogram of the frequency of samples that had otoliths aged by either (**A**) daily or (**B**) annual increments.

The daily ageing from otolith reading ranged from 7 to 633 days (Fig. 1A) and 0.4 to 15 years for the annual ages (Fig. 1B). The otolith age of each fish used in this study is provided in Supplementary Table 1.

### Sequencing data set

On average, 901,000 reads were aligned to the golden perch targeted amplicons with an alignment rate of 89.6%. All amplicons were amplified. The amplicon mean read coverage was 29,065 reads and did not correlate with the absolute error rate in the universal model (Pearson correlation = − 0.01, p-value = 0.67). The bisulfite conversion rate was predicted to be > 99% as non-CpG site cytosines were found to be converted^53,54^. All targeted 31 CpG sites that are conserved between zebrafish and golden perch were successfully amplified. An additional 18 neighbouring CpG sites within the amplicons were amplified and had methylation levels across all samples. This makes a total of 49 CpG sites for each model. An elastic net regression model was used to develop a separate clock for each comparison (Universal Clock, Daily Age Clock, and Annual Age Clock).

### Universal clock

**Figure 2:**
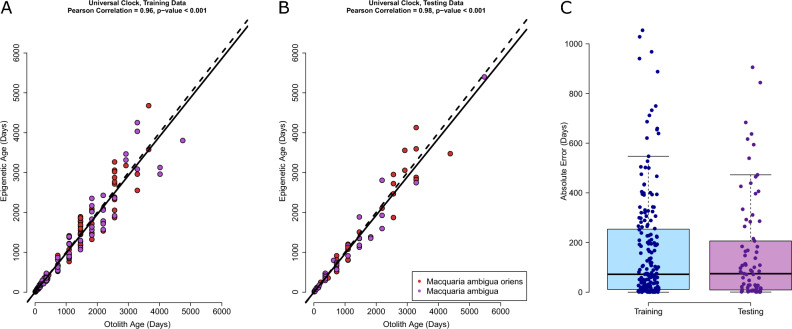
The golden perch Universal Clock calibrated with both daily and annual aged samples. Correlation between the otolith age and the predicted age in the (**A**) training data set and (**B**) the testing data set. The continuous line is the regression line and the dashed is a 1:1 line between each axis. (**C**) The absolute error rate in both the training and testing data set. The median is represented by the thick horizontal black line in each box plot.

In total, the elastic net regression model selected all 49 CpG sites across the targeted amplicons to make the final model (Supplementary Table 7). Although, the target was 31 from genome pairwise alignments, neighbouring CpG sites were found to be predictive of age. The Universal model was found to have an overall high correlation between the otolith and predicted age in both the training data set (Pearson correlation = 0.96, p-value < 0.001, Fig. 2A) and the testing data set (Pearson correlation = 0.98, p-value < 0.001, Fig. 2B). The median absolute error was 72.1 days and 74.5 days in the training and testing data sets, respectively, and no statistical difference was found between the two (paired t-test, p-value = 0.77; KS test, p-value = 0.69, Fig. 2C). The similarity in correlations and the lack of difference in overall absolute error rate suggests the model was not overfitted. The relative error in the testing data set was found to be 14% and did not correlate with increasing age, suggesting the model is not biased to a specific age group (Pearson correlation = 0.08, p-value = 0.17).

### Daily and annual clocks

The daily clock was constructed with samples ranging from 7 up to 633 days of age (Supplementary Fig. 2). The elastic net was used to generate a separate model and it selected 41 CpG sites (Supplementary Table 7). These 41 CpG sites included the 31 CpG sites conserved between zebrafish and golden perch. As described above for the universal model, a high correlation was observed between otolith and predicted age in both the training (Pearson correlation = 0.94, p-value < 0.001) and the testing data sets (Pearson correlation = 0.96, p-value < 0.001). The median absolute error was 3.5 days and 2.4 days in the training and testing data sets, respectively, with no statistical difference between the two data sets (paired t-test, p-value = 0.89; KS test, p-value = 0.34). The overall relative error for the daily age model was found to be 7.6%.

The annual clock was constructed with the samples that had annual age estimates from otoliths ranging from 0.4 to 15 years. The elastic net model selected all 49 CpG sites (Supplementary Table 7). Whilst these were annual ages, the units were converted to days for comparison with the other two models developed in this study (Supplementary Fig. 3). The annual age model yielded a high correlation between otolith and predicted age in both the training (Pearson correlation = 0.96, p-value < 0.001) and the testing data sets (Pearson correlation = 0.97, p-value < 0.001). The median absolute error was 128.3 days and 184.6 days in the training and testing data sets, respectively, with no statistical difference between the two data sets (paired t-test, p-value = 0.81; KS test, p-value = 0.75). The performance of all three models for different age groups are detailed in Table 1.

**Table 1:** Performance of each model for various age groups.

Model	Age range (days)	Sample size	Pearson’s correlation	Median absolute error rate (days)	Median relative error
Universal	< 100	19	0.96	5.1	0.20
Universal	≥ 100	62	0.97	108.9	0.13
Universal	< 1000	45	0.97	11.9	0.17
Universal	1000–2000	19	0.72	113	0.078
Universal	2000–3000	10	0.72	330.6	0.14
Universal	> 3000	7	0.79	464	0.14
Daily	< 100	19	0.98	1.7	0.069
Daily	≥ 100	5	0.98	26	0.15
Annual	< 1000	21	0.87	130	0.21
Annual	1000–2000	19	0.54	284	0.11
Annual	2000–3000	10	0.31	389	0.20
Annual	> 3000	7	0.64	741	0.21

The performance statistics are from the testing data sets.

No statistical difference was found with the training data sets (t-test and KS-test).

## Discussion

In this study we successfully developed three epigenetic clocks to estimate daily and annual ages of golden perch. By developing separate epigenetic clocks for daily and annual ages we can increase the precision depending on the age range (Table 1). We show that epigenetic clocks can be developed as fit for purpose tools to target specific knowledge gaps in wildlife management.

Age structure of fish populations is critical for monitoring, evaluation, and reporting programs for wildlife management. In 1999, Campana and Thorrold^55^ estimated more than 800,000 otoliths were being aged worldwide^56^. This included both species that are threatened and subject to unsustainable commercial exploitation. An advantage of an epigenetic clock is that it can be a non-lethal method to age prediction. Otolith ageing on the other hand, is lethal and depending on the species, can be difficult to extract and read. DNA sequencing has also become more cost effective, making it a more attractive method for wildlife management^57^. These advantages are ideal to better understand drivers of variation in larvae and daily growth rates.

Daily ageing of otoliths can be time-consuming and difficult. Multiple readers are often used to increase the precision of age estimation. However, the daily epigenetic clock provides an alternative to predict age for young-of-year fish. Using daily increments for calibration, improved the precision of the epigenetic clock, as it reduces any age uncertainty with annual ages. For example, the annual age may be 3 years, but the fish may be 3.25 years old. Therefore, that 0.25 years of information would be lost. Ideally, a study between daily and annual ages from the same fish could give insight into how the daily ages, increase the precision of an epigenetic clock, but this is outside the scope of this study. Another factor of having daily ages is that it improves the precision of a Von Bertalanffy growth curve. Accurate ages are important for juveniles and can assist in the understanding of environmental impacts on growth and spawning. More precise epigenetic clocks are essential to better understand if factors such as temperature influence the epigenetic age prediction. Temperature has been shown to have an effect on growth^58^ and may influence the epigenetic clock performance, however this has not been fully investigated^11^. More broadly, the benefit of being able to predict age more readily can lead to the better understanding of flow-ecology linkages and using these to adaptively manage human impacts (e.g. flow alteration, fragmentation, over-fishing) and interventions (e.g. fish stocking and habitat enhancements)^59^.

Ideally, individuals with a known date of birth are best suited to calibrate epigenetic clocks. Unfortunately, the species that are in the most need of epigenetic clocks, often do not have an adequate sample size for calibration. Ideally, a minimum of 220 samples is required to develop an epigenetic clock for a species with a single tissue type to minimise error rates^60^. Large sample sizes if often a barrier for many species to develop an epigenetic clock. However, some epigenetic clocks have been developed with fewer samples, and have acceptable error rates for their required downstream applications^61,62^. Alternatives to known-age samples to calibrate epigenetic clocks have included bomb radiocarbon dating, otoliths, and estimations from age-length relationships^5,62^. In this study, otoliths ageing was used to obtain a daily and an annual age estimate. Although the performance of all three models were within range of previous fish studies^3,5^, there is a limitation with otoliths. For example, fish aged by counting annual increments on the otolith lack resolution compared to ageing daily growth increments. This can increase the error rate in the model and therefore how they were calibrated should be considered when using the epigenetic clocks in future research. Interestingly, the models in this study had a higher correlation in the testing data sets. Unlike previous studies where the training data is has a higher correlation^4,63^. In this study this may be due to have slightly older samples randomly being selected into the testing data set. For example, the daily clock had a much older sample which may be skewing the correlation. However, as stated in the results there is no statistical difference between performance in the training and testing datasets. If known-age samples become available in the future, the models can always be tested against such samples or be used to improve the model.

## Conclusion

Our research further demonstrates the potential of molecular based methods for wildlife management and has a similar performance to other work in other fish species^3,5^. Our epigenetic clocks enable ageing of large sample sizes with a non-lethal and cost-effective method, which is of great benefit to a species vulnerable to anthropogenic impacts including climate change. The cumulative effects of high cultural, economic, and social value for this species, coupled with the alteration and regulation of their critical habitats has affected all aspects of golden perch ecology contributing to long-term population viability. Having a non-lethal method to predict the age of golden perch will help support fisheries management and inform ongoing conservation efforts.

## Supplementary Information

Supplementary Information 1.

Supplementary Information 2.

Supplementary Information 3.

## Data Availability

All raw data used in study are provided online on the CSIRO Data Access Portal at: https://data.csiro.au/collection/csiro:55966.
