# Supplementary material for: Accurate, non-destructive, and high-throughput age estimation for Golden perch (Macquaria ambigua spp.) using DNA methylation
Source: Sci Rep. 2023 Jun 12;13:9547. doi: 10.1038/s41598-023-36773-2 (PMC10260977; doi:10.1038/s41598-023-36773-2)
Supplement: Supplementary file 2 — Supplementary Information 2. [file 41598_2023_36773_MOESM2_ESM.docx]

Supplementary Information for Accurate, non-destructive, and cost-effective age estimation for Golden perch (*Macquaria ambigua spp.*) using DNA methylation.

Benjamin Mayne, Tom Espinoza, David A. Crook, Chloe Anderson, Darren Korbie, Jonathan C. Marshall, Mark J. Kennard, Doug J. Harding, Gavin L. Butler, Brien Roberts, Josh Whiley, and Sharon Marshall

**Contents**

| **Section** | **Page Number** |
| --- | --- |
| **Supplementary Methods** | 2 |
| Supplementary Table 1. Sample Information | 2 |
| R script | 2 |
| Bisulfite Treatment Protocol | 3 |
| Supplementary Table 2. Primer Sequences | 5 |
| Supplementary Table 3. Multiplex PCR mastermix. | 7 |
| Supplementary Table 4. Cycling conditions for multiplex PCR. | 8 |
| Supplementary Table 5*.* Mastermix to ligate barcodes | 8 |
| Supplementary Table 6. Cycling conditions for ligating barcodes. | 8 |
| **Supplementary Results** | 9 |
| Supplementary Table 7. Epigenetic Clock Model | 9 |
| Supplementary Figure 1. Images of otoliths from daily and annual golden perch | 11 |
| Supplementary Figure 2. Daily Clock Performance | 12 |
| Supplementary Figure 3. Annual Clock Performance | 13 |
| **References** | 14 |

**Supplementary Methods**

Supplementary Table 1 is provided in a separate spreadsheet for a better fit of all the data.

**Supplementary Table 1.** Sample information and age prediction of the golden perch used in this study.

**R Script.** Example R script using the Universal Age model as an example from reading the data files from Bismark to using the elastic net and the performance statistics.

**Bisulfite Treatment Protocol**

This protocol is for 150ng of DNA in 6μL

**Prerequisites and reagents required prior to starting**

| **Reagent** | **Notes/Concentrations** |
| --- | --- |
| Solid-phase reversible immobilization (SPRI) magnetic bead mix | Solid-phase reversible immobilization (SPRI) magnetic bead mix. A full protocol to make SPRI bead mix is publicly available at <https://openwetware.org/wiki/SPRI_bead_mix> |
| Low TE Buffer | 10mM Tris-HCl, pH 8.0 and 0.1mM EDTA |
| Sodium Hydroxide | 10M and 3M |
| Sodium Acetate | 3M, pH = 5.2 |
| Ethanol | 70% |

**Preparation of working solutions**

1. Prepare saturated sodium metabisulfite
   1. In a **15mL** tube weigh **4.05g** of **sodium metabisulfite** (Sigma-Aldrich, Cat: S9000-500G)
   2. Add approximately **6mL** of **Milli-Q Water**
   3. Add **247.5μL** of **10M** of **NaOH**
   4. Add **Water** to the **total volume** is **8mL**
2. Prepare hydroquinone solution
   1. In a **50mL** tube weigh **0.55g** of **hydroquinone** (Sigma-Aldrich, Cat: H9003-100G)
   2. Add **Water** to a **total volume** of **50mL** (this creates a **100mM hydroquinone** solution)
   3. In a **15mL** tube transfer **1mL** of the **100mM hydroquinone solution** and add **9mL** of **Milli-Q Water** (this creates a **10mM hydroquinone** solution)

**Bisulfite Treatment**

1. Add **0.66μL** of **3M NaOH** to each DNA sample and mix well
2. Incubate the samples using the following temperatures and time below

| Temperature (^o^C) | Time (mins) |
| --- | --- |
| 37 | 15 |
| 90 | 2 |
| 4 | Hold |

1. Place the samples on ice
2. Add **69.33μL** of the **saturated sodium metabisulfite** solution to each DNA sample
3. Add **4μL** of the **10mM hydroquinone** solution to each DNA sample
4. Mix DNA samples well by pipetting up and down
5. Incubate the samples using the following temperatures and time below

| Temperature (^o^C) | Time (mins) |
| --- | --- |
| 85 | 50 |
| 4 | Hold |

1. Add **2.5μL** of **3M sodium acetate pH 5.2** to each DNA sample
2. Add **30μL** of **Water** to each DNA sample
3. Add **175μL** of the **SPRI DNA Bead mix** to each sample
4. Mix DNA samples well by pipetting up and down and incubate for **10mins** at **room** **temperature**
5. Using a magnetic plate, separate the beads from the solution and remove the supernatant
6. Wash the DNA sample **twice** with **200μL** of **70% Ethanol** and make sure there is no ethanol left in the DNA samples prior to the next step
7. Resuspend the beads in **16.6μL** of **Low TE Buffer**
8. Add **1.8μL** of **3M NaOH** to each DNA sample and mix well
9. Incubate the samples using the following temperatures and time below

| Temperature (^o^C) | Time (mins) |
| --- | --- |
| 37 | 15 |
| 4 | Hold |

1. Add **3.6μL** of **3M sodium acetate pH 5.2** to each DNA sample
2. Using a magnetic plate, separate the beads from the solution and remove the supernatant
3. Wash the DNA sample **twice** with **200μL** of **70% Ethanol** and make sure there is no ethanol left in the DNA samples prior to the next step
4. Resuspend the beads in either **6μL** of **Low TE Buffer** or the required amount of the PCR mastermix and incubate at room temperature for **10mins**.
5. Place the plate on the magnetic plate and separate the supernatant into a new plate.

**Supplementary Table 2.** Sequences of primers tested and included in the multiplex PCR assay.

| **Included in Multiplex?** | **Chromosome** | **Position** | **Amplicon Size** | **# of CpGs** | **Forward Primer** | **Reverse Primer** |
| --- | --- | --- | --- | --- | --- | --- |
| Yes | VMKM01000962.1 | 182408 | 145 | 10 | cagagacttggtctTCCATCNATAACCTCACCC | gacatggttctacaTTATNGTAGATTTGTTAGTTTTTTTATTTT |
| Yes | VMKM01000486.1 | 94226 | 125 | 8 | gacatggttctacaCCNATACACCCCTCCTTA | cagagacttggtctTTGAGTTTAGGAGTTTTGAGTTG |
| Yes | VMKM01002175.1 | 39457 | 128 | 8 | gacatggttctacaGTNGATGATGTTTTTTATTAGTAG | cagagacttggtctAATCCAAAAACCCCTACCAC |
| Yes | VMKM01000780.1 | 239657 | 154 | 2 | gacatggttctacaCACTCTTATAATATATATATTTACCC | cagagacttggtctGAGAAGTATGTAAGAAAAGATGA |
| Yes | VMKM01003406.1 | 22109 | 137 | 4 | cagagacttggtctCACTCCNCCTCCCCTT | gacatggttctacaATNGTTGTTGGATTTTATTTTTATG |
| Yes | VMKM01000048.1 | 222503 | 143 | 15 | cagagacttggtctGTGNGTTTTTAGGATGTGATA | gacatggttctacaTAACCCNCTTCCTCTAATC |
| Yes | VMKM01000648.1 | 204246 | 150 | 7 | gacatggttctacaCCAAATACATAACCCAAACCC | cagagacttggtctAGAGTNGGTTTTTGTATTATTTG |
| No | VMKM01000177.1 | 299574 | 151 | 3 | cagagacttggtctAAACAAACTTATAATTAAAATCCTCTTAAT | gacatggttctacaGAGTAGTTAGAGAATGAGTTG |
| Yes | VMKM01000049.1 | 618153 | 144 | 5 | cagagacttggtctTTAATTAGNGTTTTGATGATAGTTG | gacatggttctacaCCCTATAATCNCAAACTATTAC |
| Yes | VMKM01000291.1 | 71235 | 149 | 2 | cagagacttggtctGTTTTTATGTGTATTTGTAAGGTAT | gacatggttctacaTTTTAAACACTTAATTAAATATCCATCTATT |
| Yes | VMKM01000094.1 | 117171 | 153 | 4 | gacatggttctacaTCNCACTTATAAAACTTCTCC | cagagacttggtctGTTTTTTTAGTTATTTTATTTGTTTTATTATTAG |
| Yes | VMKM01003526.1 | 28334 | 139 | 7 | gacatggttctacaTAGGTTGTNGTTTTGGTTGAA | cagagacttggtctAATCNACAACACCTACCAC |
| Yes | VMKM01000284.1 | 95241 | 154 | 2 | gacatggttctacaACCACCACTTCAATAACCC | cagagacttggtctGAGGAAAGGATTGTGAATGAA |
| Yes | VMKM01000223.1 | 197591 | 125 | 2 | gacatggttctacaTATTTTTAGTTTTGTGTGTTTTGAG | cagagacttggtctCTCCNTATCCACATCCAAT |
| Yes | VMKM01000049.1 | 167182 | 141 | 5 | gacatggttctacaTCAAACTTCNATTTAAATCTAATCA | cagagacttggtctGGAAATGANGGTAGTTAGG |
| Yes | VMKM01001960.1 | 73838 | 148 | 4 | gacatggttctacaCCNTTATCTTTCACTACCAC | cagagacttggtctANGTTTGGTTGTTTTATTATATAGT |
| Yes | VMKM01000004.1 | 1220241 | 144 | 5 | cagagacttggtctCTAACCCNATAATTCCCTTAT | gacatggttctacaTGTTAGTAGAATAAGTTAGTTTAGT |
| No | VMKM01000223.1 | 292211 | 125 | 2 | gacatggttctacaCATATAACNCCCCCCAAAA | cagagacttggtctTGAGTTATTTTTTAATGTATATTTTTATTTTTTA |
| No | VMKM01001600.1 | 126869 | 150 | 5 | gacatggttctacaAAGGTGATTGTTTAGATAGTATAAT | cagagacttggtctCCTACNACAAAACCTATTCC |
| No | VMKM01000899.1 | 14283 | 153 | 4 | gacatggttctacaCAAAAAACCCCAAACATAATTTAC | cagagacttggtctGTTTGTTAATTGTATTGTTTAGATATATGA |
| Yes | VMKM01000925.1 | 34850 | 131 | 4 | cagagacttggtctATAATCCAATAATAACCTTTCCC | gacatggttctacaNGTTTTTTATTTTTTAGAGGTTTATATTAG |
| Yes | VMKM01001684.1 | 114470 | 154 | 5 | cagagacttggtctAAAACTCTCTACCCACCAC | gacatggttctacaNGGTTTTTGAATATAAATAGTTAGG |
| Yes | VMKM01001157.1 | 4858 | 143 | 4 | gacatggttctacaGATAGNGTGTTATTTTATATTAGG | cagagacttggtctCTTAATATCTCTATTATCTTCCC |
| Yes | VMKM01000191.1 | 413504 | 149 | 3 | gacatggttctacaGATGAGGAGTTTTAATATAATTGAG | cagagacttggtctTATTCTCCAATCTATTAAATCATTTAATAT |
| Yes | VMKM01000295.1 | 173841 | 146 | 3 | cagagacttggtctAAACCCATATATACTCCTCATTA | gacatggttctacaGTTTATAGTATGATATAAGATTTGAAATAT |
| Yes | VMKM01000511.1 | 278923 | 153 | 3 | cagagacttggtctTTAANGGTATTTTGATTGTTGTTAG | gacatggttctacaTCATCCAAAAACTACCTACAC |
| No | VMKM01000776.1 | 38360 | 155 | 3 | cagagacttggtctGTAANGATTGTGAAATTATTTTATTTTTAT | gacatggttctacaTCCTTTAACTACTCCTCTTTAAT |
| No | VMKM01003432.1 | 7546 | 144 | 2 | cagagacttggtctATTTATTTGGTTTTATTAGTTTAAAAATTATTAG | gacatggttctacaTCAAATTCTAAATATATATCTAAATCAAAATT |
| Yes | VMKM01000633.1 | 125563 | 128 | 3 | gacatggttctacaACCTCTACACCTACTTCAC | cagagacttggtctGTTGTTAGAATAAAAGTAGTTGAG |
| Yes | VMKM01000604.1 | 273842 | 142 | 2 | gacatggttctacaTTTGTTTTTTTTATAATTTTAGTTTATGTAAGAA | cagagacttggtctTAACCCNCATCATAAACCC |
| No | VMKM01000273.1 | 182004 | 144 | 2 | cagagacttggtctAATTAAATTCTTTTTACAATAAAAACCAATAT | gacatggttctacaNGTTTTTTTTGATTTTTTTGATTTATATTATG |

**Supplementary Table 3.** Multiplex PCR mastermix. The reaction details are for a 50μL reaction. The Combinatorial Enhancer Solution (CES) solution recipe was obtained from a previous study [1].

| **Reagent** | **Company** | **Catalogue No.** | **Stock Concentration** | **1x Reaction (μL)** |
| --- | --- | --- | --- | --- |
| 5X Green GoTaq® Flexi Buffer | Promega | M891A | 5x | 10 |
| GoTaq® Hot Start Polymerase | Promega | M500A | 5U/μL | 0.25 |
| MgCl_2_ | Promega | A351B | 25mM | 9 |
| Combinatorial Enhancer Solution (CES) | See below | NA | 5x | 5 |
| dNTP | Fisher Biotec | DN-10M-10 | 10mM | 0.5 |
| TMAC | Sigma-Aldrich | T3411-500ML diluted to 1M | 1M | 0.75 |
| Primers | IDT | - | 0.8μM | 12.5 |
| Water | Sigma-Aldrich | W4502-1L | - | 7 |
| DNA | - | - | Final < 2ng/μL | 5 |
| ***Combinatorial Enhancer Solution (CES)* Recipe** | | | | |
| **Reagent** | **Company** | **Catalogue No.** | **Stock Units** | **5000μL Stock** |
| Betaine | Sigma-Aldrich | B0300-5VL | 5M | 2700μL |
| DTT | Thermofisher | P2325 | 1M | 33.5μL |
| Dimethyl Sulfoxide (DMSO) | Sigma-Aldrich | D2650-5X5ML | 78.13g/mol | 335μL |
| BSA | New England Biolabs | B9000S | 20mg/mL | 13.75μL |
| Water | Sigma-Aldrich | W4502-1L | - | 1917.75μL |

**Supplementary Table 4.** Cycling conditions for multiplex PCR.

| **Step** | **Temperature (°C)** | **Time (seconds)** | **Cycles** |
| --- | --- | --- | --- |
| Initial denaturation | 94 | 300 | 1 |
| Denaturation | 94 | 20 | 28 |
| Annealing | 56 | 30 |  |
| Extension | 72 | 120 |  |
| Final extension | 72 | 300 | 1 |
| Hold | 4 | Hold | 1 |

**Supplementary Table 5*.*** Mastermix to ligate barcodes for pooling samples prior to DNA sequencing. The total reaction size is 30µL.

| **Reagent** | **Company** | **Catalogue No.** | **Stock Concentration** | **1x Reaction (μL)** |
| --- | --- | --- | --- | --- |
| 5X Phusion Green HF Buffer | Thermofisher | F537L | 5x | 5 |
| Phusion Hot Start II High-Fidelity DNA Polymerase (2 U/µL) | Thermofisher | F537L | 2U | 0.25 |
| MgCl_2_ | Thermofisher | F537L | 50mM | 3.6 |
| dNTP | Fisher Biotec | DN-10M-10 | 10mM | 0.6 |
| Water | Sigma-Aldrich | W4502-1L | - | 5.55 |
| Fluidigm Barcode | Fluidigm | 100-4876 | 0.8μM | 5 |
| DNA | - | - | PCR Product | 10 |
|  |  |  | Total | 30 |

**Supplementary Table 6.** Cycling conditions for ligating barcodes.

| **Step** | **Temperature (°C)** | **Time (seconds)** | **Cycles** |
| --- | --- | --- | --- |
| Initial denaturation | 94 | 300 | 1 |
| Denaturation | 97 | 15 | 12 |
| Annealing | 45 | 30 |  |
| Extension | 72 | 120 |  |
| Final extension | 72 | 120 | 1 |
| Hold | 4 | Hold | 1 |

**Supplementary Results**

**Supplementary Table 7.** Genomic coordinates and corresponding coefficients for each model.

| **Chromosome** | **Position** | **Universal Age Model** | **Daily Age Model** | **Annual Age Model** |
| --- | --- | --- | --- | --- |
| (Intercept) | NA | 4.131413333 | 3.875021815 | 6.659791404 |
| VMKM01000004.1 | 1219607 | 0.000534092 | 2.34115E-39 | 7.55502E-05 |
| VMKM01000012.1 | 223829 | 0.00226851 | 2.68413E-39 | 0.000397474 |
| VMKM01000012.1 | 224124 | 2.27528E-08 | -1.13182E-38 | 0.000309584 |
| VMKM01000012.1 | 224258 | 0.001150721 | NA | 0.00064628 |
| VMKM01000017.1 | 181031 | 0.00233504 | -3.03124E-39 | 0.000833507 |
| VMKM01000048.1 | 221350 | 0.000943928 | -1.2614E-40 | -6.76088E-05 |
| VMKM01000049.1 | 617032 | 0.002115258 | 4.44506E-39 | -2.03739E-05 |
| VMKM01000049.1 | 617084 | 0.000376423 | 1.19722E-39 | -8.66297E-05 |
| VMKM01000191.1 | 412434 | 0.002227168 | 5.10922E-40 | 0.000290533 |
| VMKM01000223.1 | 196437 | 0.000645568 | -2.5076E-39 | -0.000153626 |
| VMKM01000223.1 | 196980 | 0.000484452 | -3.12658E-39 | 0.000214189 |
| VMKM01000223.1 | 291025 | 0.000807314 | 9.41147E-39 | 0.000573141 |
| VMKM01000223.1 | 291029 | 0.001308876 | -6.36491E-39 | 0.000840139 |
| VMKM01000273.1 | 180770 | 0.001280713 | -9.18679E-40 | -0.000510807 |
| VMKM01000273.1 | 180815 | 0.000136891 | -2.81946E-39 | 8.82743E-05 |
| VMKM01000273.1 | 181485 | 0.003286169 | NA | 0.002630717 |
| VMKM01000427.1 | 113876 | 0.002109504 | NA | 0.001017534 |
| VMKM01000427.1 | 114019 | 0.000120074 | -5.21262E-39 | 0.000737856 |
| VMKM01000486.1 | 93101 | 0.018421381 | 2.99384E-38 | 0.003513176 |
| VMKM01000486.1 | 93283 | 0.006056463 | NA | 0.000918373 |
| VMKM01000486.1 | 92789 | 0.001751876 | -1.12601E-39 | 0.000664147 |
| VMKM01000511.1 | 277999 | 0.000940385 | 2.82263E-39 | 0.000131091 |
| VMKM01000511.1 | 278107 | -0.002873753 | -1.06958E-38 | 0.001719751 |
| VMKM01000513.1 | 188298 | 0.000253666 | -1.81523E-39 | -2.27423E-05 |
| VMKM01000513.1 | 188351 | 0.000387011 | 2.94253E-39 | 0.000244499 |
| VMKM01000586.1 | 71168 | 1.6078E-05 | -3.57957E-39 | 0.000259828 |
| VMKM01000648.1 | 203225 | 0.001605686 | -4.20197E-39 | 0.001454322 |
| VMKM01000648.1 | 203611 | 0.007423603 | 3.89616E-39 | -0.00011201 |
| VMKM01000776.1 | 37280 | 7.09363E-05 | -1.40981E-39 | -0.000210607 |
| VMKM01000899.1 | 13215 | 0.001625104 | -1.10246E-39 | 0.000260577 |
| VMKM01000925.1 | 33469 | 0.003453169 | NA | 0.0013032 |
| VMKM01000925.1 | 33663 | 0.005478601 | NA | 0.001523723 |
| VMKM01000925.1 | 33691 | 0.0009768 | 3.35151E-39 | -4.27276E-05 |
| VMKM01000962.1 | 181527 | 0.000679039 | 1.85413E-39 | 8.65623E-05 |
| VMKM01000962.1 | 181605 | 0.004333373 | NA | 0.001443159 |
| VMKM01000962.1 | 181701 | 0.002472889 | 9.62129E-40 | 0.000673684 |
| VMKM01001157.1 | 4181 | 0.005356841 | 7.86998E-39 | 0.000903283 |
| VMKM01001157.1 | 4205 | 0.000665734 | 5.89643E-40 | 0.000704084 |
| VMKM01001290.1 | 139231 | 0.004783886 | 8.89378E-40 | 0.000833101 |
| VMKM01001359.1 | 75878 | 0.001719645 | 1.86734E-39 | 0.000608729 |
| VMKM01001600.1 | 126229 | 1.23113E-05 | -4.48781E-39 | 0.000291736 |
| VMKM01001684.1 | 113120 | 0.004331271 | -3.95167E-39 | 0.000755962 |
| VMKM01001960.1 | 72390 | 0.000287757 | -7.60976E-40 | 0.000851249 |
| VMKM01002175.1 | 38920 | 0.002669212 | -3.79007E-39 | 0.000359605 |
| VMKM01003432.1 | 6324 | 0.000429842 | NA | 9.24846E-05 |
| VMKM01003526.1 | 27219 | 0.00075532 | 2.17936E-39 | 0.001325437 |
| VMKM01003526.1 | 27388 | 0.004933947 | -3.66411E-39 | 0.000132267 |
| VMKM01003526.1 | 27493 | 0.000877595 | 1.94537E-39 | 0.000233191 |
| VMKM01003526.1 | 27510 | 0.001494408 | 1.15406E-39 | 0.000385184 |


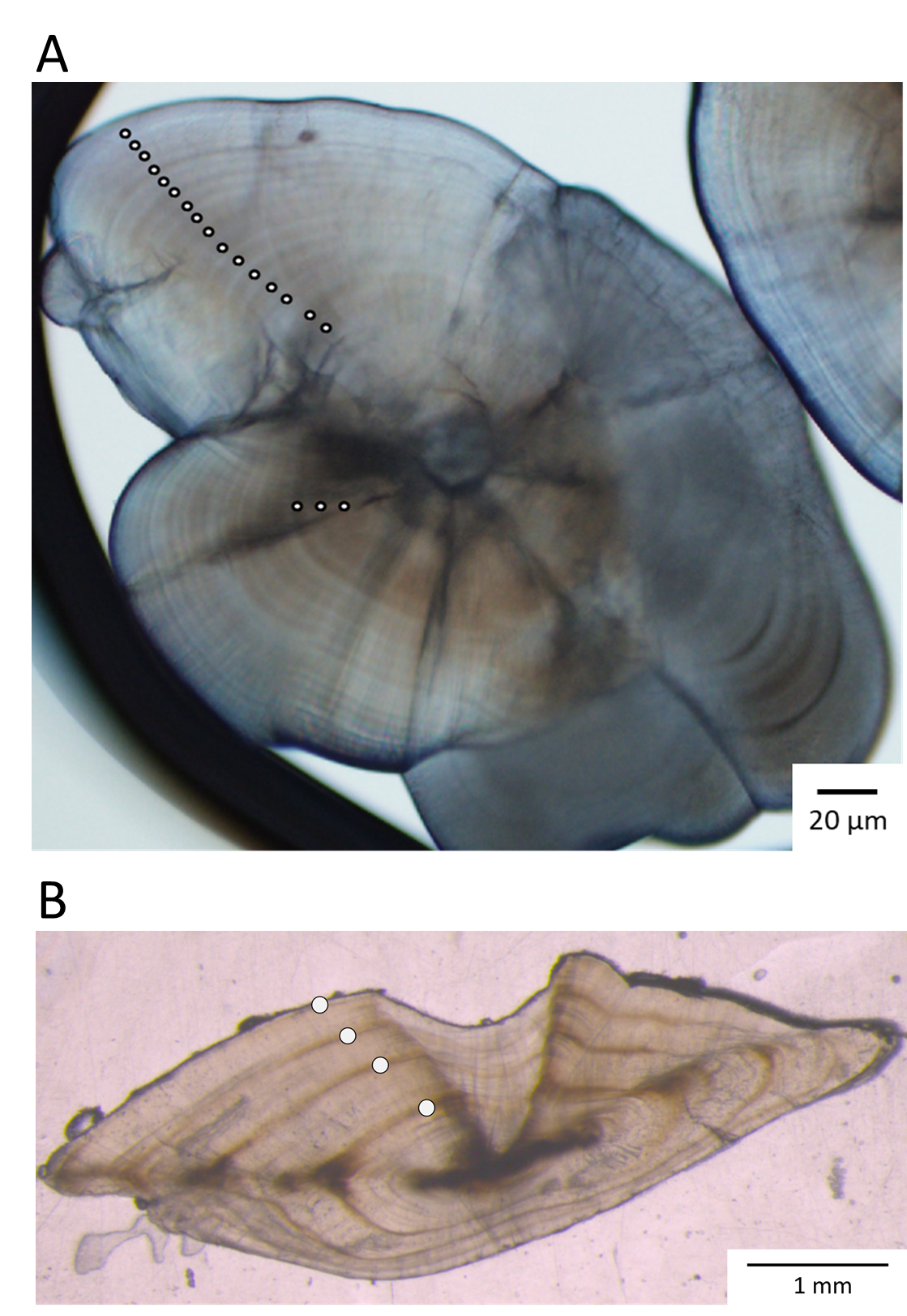
**Supplementary Figure 1. A**. Image of an otolith from a 14 mm (SL) juvenile golden perch with 19 daily increments marked, corresponding to an estimated 24 days of age (photograph by Josh Whiley). **B**. Image of an otolith from a 253 mm (SL) adult golden perch with 4 annual increments marked (photograph by Brien Roberts).


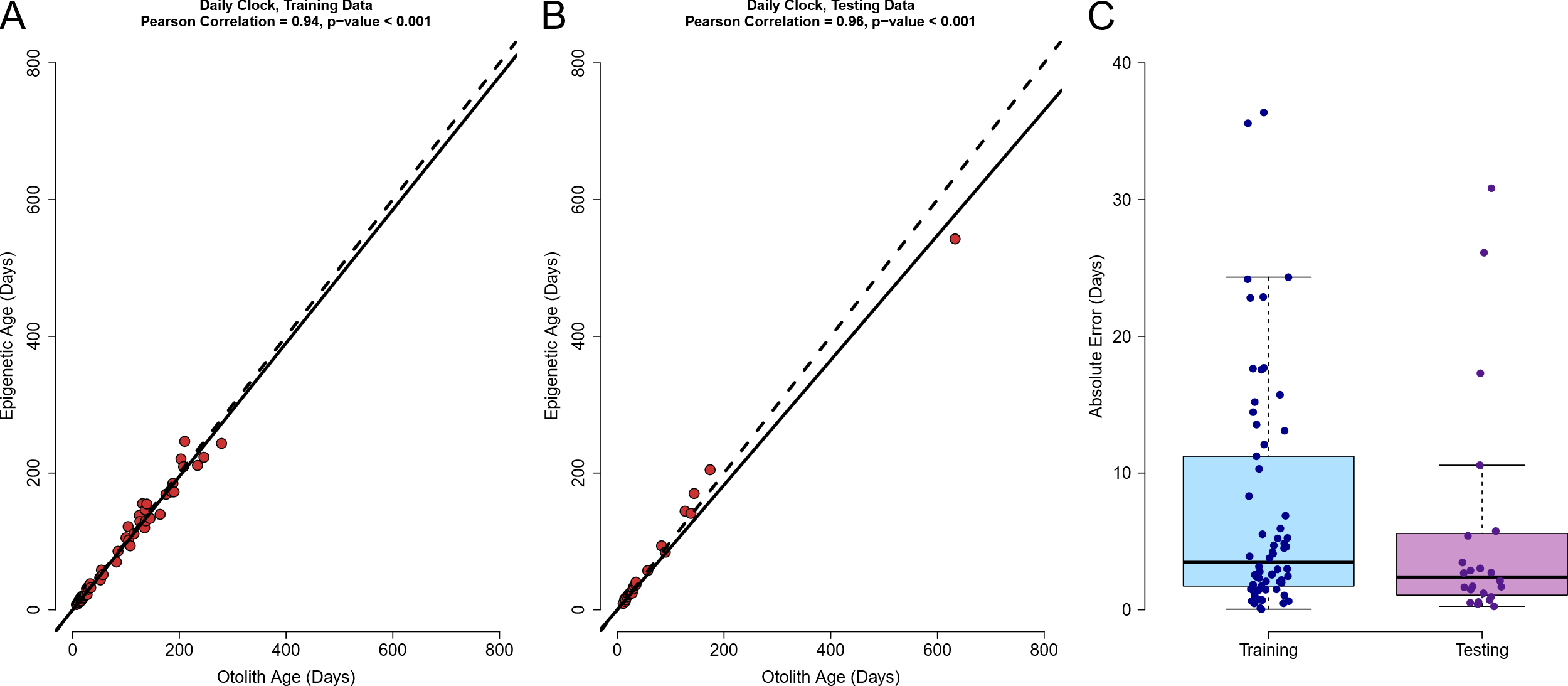


**Supplementary Figure 2.** The golden perch daily clock calibrated with only samples that had otoliths aged into days. Correlation between the otolith and predicted ages in **A.** training data set and **B.** the testing data set. The continuous line is the regression line and the dashed is a 1:1 line between each axis. **C.** The absolute error rate in both the training and testing data set. The median is represented by the thick horizontal black line in each box plot. Only *Macquaria ambigua* species were available for daily ageing.


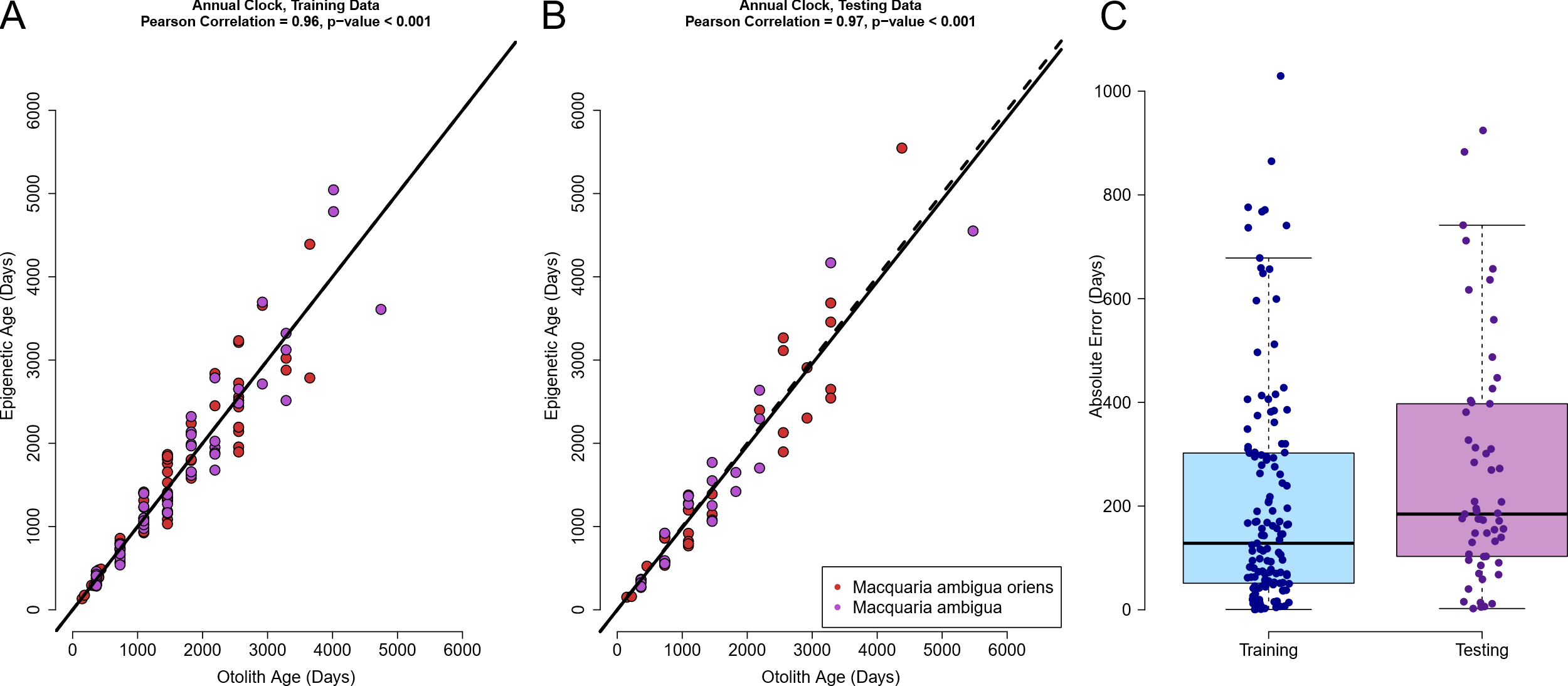


**Supplementary Figure 3.** The golden perch annual clock calibrated with only samples that had otoliths aged into days. Correlation between the otolith and predicted ages in **A.** training data set and **B.** the testing data set. The continuous line is the regression line and the dashed is a 1:1 line between each axis. **C.** The absolute error rate in both the training and testing data set. The median is represented by the thick horizontal black line in each box plot.

**References**

1. Ralser M, Querfurth R, Warnatz H-J, Lehrach H, Yaspo M-L, Krobitsch S: **An efficient and economic enhancer mix for PCR.** *Biochemical and Biophysical Research Communications* 2006, **347:**747-751.
